# Notes of Four Cases of Anasarca and Ascites with Albumen in the Urine

**Published:** 1846-06-01

**Authors:** Geo. N. Burwell


### BUFFALO MEDICAL JOURNAL

AND

### MONTHLY REVIEW.

Vol. 2. JUNE 1, 1846. No. 1.

### ORIGINAL COMMUNICATIONS.

ART. I.—*Notes of four cases of Anasarca and Ascites with Albumen in the Urine.* By GEO. N. BURWELL, M. D.

*Case* I. *Detailed Note, August* 18, 1843.—Susan Pierson, aged 28 years, unmarried, entered the Philadelphia Hospital, Blockley, July 21, 1843, with anasarca and ascites. Has always been temperate; is of middling statue; temperament sanguineous. She is now considerably emaciated and pale. She says she enjoyed excellent health until the fall of 1841, when she caught cold from exposure to the rain. Besides a cough, she had then pain and swelling of the limbs. These disappeared on the application of a liniament for a few days. She thought this to be rheumatism. At this time, also, her courses stopped, and she has never been perfectly well since. She had no return of the swelling until the fall of 1842, when, after getting her feet wet, her abdomen swelled up to nearly its present size, (that of a woman at full time) but with little or no œdema of her limbs. She took juniper-berry tea, which dissipated the swelling in about three weeks. After this she remained pretty comfortable, except suffering from rheumatic pains and general feebleness, until she got wet again during a thunder shower, about the 23rd of May last. She went to bed after drying herself, and on getting up the next morning was surprised to find her face, arms, legs and belly all swelled up. Did not have any fever. After two or three days, the œdema left her face and arms, but she has ever since had frequent turns of swelling in these parts, which last a day or two and then subside. Has had slight exacerbations of fever during the last few weeks; is obliged to make water frequently: has no bowel complaint nor cough. Pulse now 100, small; skin rather dry; never sweats without the hot bath; has pains all through her back and abdomen. Complains of shortness of breath, and of turns of feeling very weak and uncomfortable. When these pass off, she would feel as well as ever, were it not for the dropsy. Her skin is sallow. There are no signs of heart disease. The ascites prevents a satisfactory examination of the liver; abdomen tense; anasarca of the legs and thighs which pit on pressure; urine yields a copious precipitate of albumen, both on the application of heat, and the addition of nitric acid.

The ascites and anasarca both increasing at present; her respiration is short and difficult, and she has to be supported nights in a sitting posture. She is able to walk about the ward during the day.

The treatment first adopted was the exhibition of cathartics. which afforded relief for a few days; but soon they began to produce great irritation of the bowels; profuse diarrhœa followed with griping; her tongue became red, like raw beef.

All the treatment thenceforth was directed to controlling these untoward symptoms. The diarrhœa proved very obstinate, and she became so much reduced by September that she was able to walk only a few feet.

About the middle of September, gangrene of one of her legs set in, and extended nearly to the knee. This afforded an outlet to the water, and all the anasarca and most of the ascites soon disappeared, changing her appearance in an astonishing manner. This gangrene was spontaneous, and not brought on by any attempts at scarification or acpuncturation of the limb. She lingered in this horrible condition for a week and then died. There was a post-mortem examination, but much to my regret, I neglected at the time to make a note of it. I will not, therefore, say more of it than that the kidneys were the only organs diseased, and they presented what all of us who saw them considered to be an excellent specimen of "Bright's disease of the Kidney."

This case illustrates very well the common mode of accession, progress and the result of ordinary cases of the chronic form of Bright's disease, or, as frequently called, Granular Degeneration of the Kidneys.

While in a state of apparent good health the patient is attacked with general anasarca after exposure to wet and cold, accompanied with more or less fever, rapidity of pulse, &c., as the attack partakes of acute disease, or approaches more to an acute or a chronic form. By prompt and judicious treatment the fever is subdued, the anasarca removed, and the patient is again apparently well. But this proves to be deceitful in a large majority of cases, especially if the first attack partook of the sub-acute or chronic form. There is then a renewal of the anasarca after an interval of some weeks or months. Again it may be removed, and the patient thinks himself well, except, perhaps in suffering some from rheumatic pains, until his hopes are again dashed by a renewal of the dropsy. Finally, after these repeated attacks at longer or shorter intervals, for months, or years even, he finds himself hopelessly dropsical and destined to die a miserable death, unless his life be sooner cut short by some intercurrent disease. The most common termination of this disease of the kidneys, is by coma of some days duration, before death finally closes the scene. The post-mortem examination reveals disease of the kidneys only, or a complication of kidney disease with disease of the heart and liver, known to pathologists as the "triple lesion."

Although there is experienced in this case, the usual difficulty of determining the precise period of the commencement of the organic change in the kidneys from which the dropsy resulted, yet it is interesting to note how each attack of dropsy was more severe than the one preceeding it. The first one amounted merely to an œdema of the limbs. In the second, there was the more severe form of ascites; while in the the third there was general swelling of the face and extremities, and ascites combined, and which proved fatal in four months. The probabilities are certainly very strong that this regular increase in the severity of each attack denoted a corresponding increase of the disease of the kidneys, and to my mind, also, it is very probable that the diseased condition of these organs had reached a certain stage before the first attack in 1841. But this is a point not proved.

It will be noticed that the second attack, (in the fall of 1842,) was principally ascites. This is an exception to the common course of kidney disease. The sudden supervention of anasarca ought always to lead us to suspect and search for this disease of the kidneys, while in an attack of ascites we look elsewhere for the cause. This is the rule. I find a similar exception to this law in the Bulletin of Medical Science for June 1844, copied from the "Proceedings of the Pathological Society of Dublin," and of which I will here make an abstract. The case was that of a young girl, six years of age, who had become affected with ascites after scarlatina. "The ascites was large and well marked, while there was but a slight degree of anasarca, and that limited to the lower extremities. There was great difficulty of breathing, and a severe pain in the left hypochondrium, which caused the child to scream violently and frequently. Under these symptoms she gradually sank, and for seven or eight days before death, was completely comatose. It was thought that some relief might have been obtained by paracentesis of the abdomen, but to this operation the child's family would not consent. When the body was examined after death it was found that the abdomen contained a large quantity of straw-colored fluid, with which the peritoneum was distended and the viscera forced from their natural situations. There was no lymph deposited in any part of the abdomen. The peritoneum had the appearance of having been a long time bathed in fluid. The liver was congested and its serous coat opaque. A very interesting circumstance was the existence of Bright's disease in the cortical structure of the kidneys (which was well represented in a drawing made from the recent part.) The distension of the abdomen had diminished the capacity of the chest. The lungs had been so compressed that they had the appearance of carnified lung, though they were not quite so solid, and hence the great dyspnoea so obvious in the latter stages of the illness. The remarkable features in the case were these—the occurrence of Bright's disease, with very little anasarca, and the diminished volume of the lungs, giving them, to a great degree, the appearance of carnification."

Another point of interest in the case first detailed, was the total inability of the patient to bear the exhibition of cathartics and diuretics, from the great irritation they caused in the primæ-viæ, while a vexatious diarrhœa did not reduce the dropsy. The last point of interest to which I shall draw attention, was the occurrence of gangrene and the rapid discharge of the water, not only from the cellular tissue of the body, but also from the abdomen.

*Case* II. Frederick Harvey, aged 46 years, a German, entered the hospital, January 20, 1844. Has general anasarca with ascites. He is a soap-maker; has been in this country about eighteen months; is in the habit of drinking largely of beer, and occasionally only of whiskey; never gets intoxicated.

There was at the time of his entrance considerable oppression of breathing, with a feeble first sound of the heart. He was bled with considerable relief. The day after the bleeding he had great pain in the head, for which a number of dry cups were applied. The following night he had two paroxysms of convulsions. The first notice the nurse had of them was a scream from the patient, and on going to his bed he found him insensible, frothing at the mouth, with general working of the limbs. It was half an hour before he ceased to be convulsed. The second convulsion lasted only about ten minutes. He lay after this in a state of stupor during six or seven days, from which he could not be roused to answer questions. He, however, took without difficulty his drinks and his medicines. For the convulsions he was cupped freely on his neck and temples, and had a blister on the back of the neck. During the week he lay in the state of stupor he had purges of jalap and cream of tartar daily repeated, with the occasional administration of calomel. He had also a diuretic tea of juniper-berries and cream of tartar.

He came under my charge on the first of February, and was then sensible and rapidly improving. The treatment was not changed.

Feb. 6. Able to sit up for the first time. The œdema has nearly left his face. His feet and ancles yet swollen. There remains also considerable fluid in his abdomen. The urine has been tested frequently and always gives a copious deposit of albumen.

Feb. 9. The legs just above the ancles pit moderately on pressure; ascites much diminished; appetite good; is about the ward all day; purges to be given hereafter only twice a week.

Feb. 22. Urine still slightly albuminous. He was kept some days longer in the ward, and finally discharged with all functions apparently well performed.

This case is very imperfectly taken, and is principally interesting from the occurence of convulsions, their severity, the long continued stupor which followed them, and his entire recovery without any impairment of his mind. It is a question whether a repetition of the bleeding on the commencement of the headache would not have prevented, or at last mitigated the severity of the convulsions, and shortened the period of stupor which followed. The occurence of convulsions, or coma, in cases of acute anasarca are always exceedingly dangerous, and ought to be met with great promptness and decision. The physician ought always to watch carefully for the earliest symptoms of their approach, and any complaint of headache, vertigo, dimness of, or partial vision, or any unusual sensations about the head, ought at once to excite alarm.

*Case* III. William Taylor; aged 42, laborer, intemperate for the last ten years. Entered the ward January 29, with general anasarca. Was perfectly well a week before. Attributed his sickness to exposure immediately after having had a warm bath: he was sent to the Alms House from the street, and being very filthy, was first given a good washing, and then sent to his ward. The night being very cold he suffered for want of sufficient covering to his bed. It was three days after having had the bath, before he perceived the Anasarca. This increased so much in the four following days that he applied for admission into the Hospital.

He was then purged with jalap, and had a diuretic drink of juniper berries and cream of tartar. On the second of Febuary he complained of considerable headache with vertigo: that night he had two convulsions, but with only partial loss of consciousness. He frothed at the mouth, bit his tongue, and had general spasms: these lasted about ten minutes each. He had a third one the next night. For these he was cupped freely and had a blister applied to the nuchae, with cold applications to the head as in the previous case.

Feb. 6. The purges have acted well, and his urine has been much increased in quantity by the diuretics. It has been tested a number of times since his entrance and always found to be albuminous. The œdema has left his face and limbs. There is yet considerable fluid in his abdomen. Has had considerable cough for the last four days but which is entirely bronchitic. Does not sit up yet.

Feb. 13. General condition much improved since last note. There is no œdema of any part; ascites much diminished during the last week; cough better; pulse 68.

Is to take a powder of jalap and cream of tartar every second day, and the diuretic tea daily ;—to use a linament to abdomen and to keep up constant compression and support upon it with a roller.

He was kept under this treatment the rest of the month when he was discharged as cured.

The similarity of this case to the second is very apparent, and it furnishes farther evidence of the necessity, in acute anasarca, of constant inquiry for any symptoms of brain disease, and of meeting those symptoms at once. It will not do to pass them by as of no account or meaning.

Both this and the last case are interesting on account of the simplicity of the treatment depended upon to remove the dropsy; and its success only shows how readily nature sometimes responds to our remedies when not opposed and embarrassed by extensive disease. They are in this respect much like the two first attacks of Case I; while the final result in that case should warn us of the uncertainty of any promises in these of a positive and final cure. These men passed from my notice on their discharge from the Hospital, and I cannot say whether either of them ever had a relapse.

*Case* IV. Mrs. Welch, an Irish woman, aged about 30 years; pregnant with her second child. I was called to see her March 17, 1845. She was suffering greatly from pain through her abdomen, back and limbs, and had thus suffered, she said, for three weeks, getting neither rest nor sleep, night nor day. She is of small stature; has the appearance of having twins, so large is her abdomen; thinks her time is up, but cannot say positively; legs and thighs largely anasarcous. I bled her about a pint, with immediate and great relief, which continued for four or five days. The serum of the blood drawn, was decidedly milky; that is, whitish and opaque; the clot was moderately firm; no buff. After this interval of relief had passed by, she continued to suffer greatly most of the time until her confinement. I bled her a second time from the arm, and once from the temple, on account of a difficulty in getting blood from the arm — anodynes were freely given without affording, apparently, the slightest relief: the second bleeding gave relief for one day only; the third did not afford the least.

During all this time the anasarca continued to extend, more and more rapidly I thought, after each bleeding. By March 31, it had reached the waist and shoulders. The skin over the hypogastric region became covered with large blebs of water which broke and discharged. It also commenced in the wound made in the temple, and extended thence to the left eye and side of the face The labia were also very œdematous, one of them swelling to the size of a man's wrist, rendering a per-vaginal examination difficult, and painful to her.

Labour finally commenced late in the evening of March 30, and was not completed until the afternoon of April 1. There proved to be twins. Twenty hours intervened between their delivery, and the second child was not born until after the exhibition of ergot. There was next, retention of the placentae, requiring artificial delivery. I do not propose to go farther into the history of the labour, as it would be foreign to my object.

Twelve hours after the completion of the labour there was distinct fluctuation in the abdomen, which continued to increase, while the anasarca diminished, especially in her limbs; there being an apparent change in the seat of the water from the cellular tissue to the abdomen. Her pulse which was 120 at the completion of the labour, fell in twenty-four hours to 96; the skin was warm and quite moist; respiration easy, short.

She flowed pretty freely for the first four days, remaining most of the time comfortable; urine free; bowels opened by some rhubarb; not the least appearance of any secretion of milk. On and after the fifth day the secretion of urine became scanty; the lochia stopped; the anasarca increased without any diminution of the ascites; she became feverish with a pulse of 120; and had a great deal of pain through her bowels, with constant tenesmus. The success of treatment was variable, she being better on some days and on others worse.

On the 4th of April I commenced giving her calomel in half-grain doses, hoping to affect her system by it, without producing ptyalism. I feared inflammation of a low grade about the uterus or its appendages. After taking ten grains in the course of six days without any apparent benefit, either from it or from the common cathartics and diuretics which I had administered rather freely, I determined to leave off all medicines for two or three days, except a little opium to procure rest at night.

On the 14th of April (four days after the calomel was discontinued) she began to complain of her teeth being sore. There was no sponginess of the gums, nor ptyalism, nor mercurial fetor to her breath, yet the teeth were so tender as to prevent her from biting anything of any consistence. Simultaneously with this she began to make water freely, passing from three to four quarts daily of clear, light-colored urine, with great relief. Up to this time (April 14) the urine had been tested frequently, and always gave a copious deposit of albumen.

She improved rapidly during the week following the date last mentioned. She continued to discharge freely from the kidneys. Her pulse kept rapid although she was free from fever. The only medicines she took were eight drops of Tinct. ferri chloridi, four times a day, and half a grain of opium every night.

On Saturday, April 19, and Monday, April 21, the urine was tested, but gave no precipitate of albumen—still some fluctuation in abdomen.

April 24. The rapidity of the pulse continues; there is now no evidence of any fluctuation in the abdomen; still some œdema of feet and ancles; appetite moderately good; bowels keep regular although she takes half a grain of opium every night. The tinct. ferri chloridi continued; to take a meat diet.

April 30. She sent for me on account of a severe head-ache, principally in the left temple and the occipital region. It was intensely severe; no ascites; the œdema of the feet and ancles so far reduced that she can wear her shoes. I had her cupped for the head-ache without relief. I next gave laudanum and antimony in large doses until she vomited. This relieved her. Her pulse still keeps up to 120. It was hard and corded before the vomiting. After this it became softer but still as frequent as before.

The urine was again tested, but without yielding any evidence of albumen.

She afterwards had other attacks of the headache but not as severe as the one above described. She also consulted me occasionally during the summer for rather severe neuralgic pains in her back and limbs.

I have seen her within a month. She had enjoyed uninterrupted good health for the six or eight months last past, as far as the organic functions were concerned. Her only complaint was of a headache which she still suffered from at intervals. The pains in her limbs were insignificant. Pulse 80, soft. She is again pregnant.

The above case interested me very much while under treatment. In the first place, the pain she suffered before her confinement, and the general inefficacy of the treatment, were, so far as my experience goes, very unusual. Half a grain of morphine at night, or a drachm of laudanum failed to give even temporary relief, and she complained of their giving her a sense of swelling up, meaning by it, I suppose a sense of tightness about her chest. A portion of the pain she suffered was due, I have no doubt, to the great distension of the walls of the uterus. I think I have seen other cases of lingering pain during the eighth month from the same cause.

As already stated, the first bleeding gave relief for four or five days, the second for twenty four hours only, while the third of sixteen to twenty ounces, thirty six hours before labour commenced, did not afford the slightest comfort. I have not satisfied myself how far the repeated bleedings were a cause of the increase of the anasarca, if they had any such effect; although, as mentioned, it appears as if it extended more and morerapidly after each bleeding.

The evening labour commenced I had determined to puncture the membranes, but finding signs of the dilatation of the os-uteri, of course I let the labour go on, without interfering with it.

The labour itself was accomplished only after a great deal of suffering. The first child presented the breech, and was only delivered after making strong traction for some time by hooking a couple of fingers in its groin. The second required ergot.

On finding my patient so comfortable the morning after the labour, I indulged the hope that she would have a rapid recovery. While the lochia and urinary discharge remained free she appeared to be doing pretty well, except there was not that rapid disappearance of the anasarca as is usual in simple œdema of the extremeties which so often accompanies the latter stages of gestation; while the accumulation of fluid within the abdomen gave new cause for anxiety.

There was never, in this case, any effort on the part of the system to establish the secretion of milk either at the third or fourth day, or after her recovery. But, by the time this secretion should have been fairly established, the lochia stopped, the urine became scanty, and the anasarca increased, with a general feverish state of the system.

It was on the supervention of these symptoms that I was first led to examine the urine, and found it albuminous. It remained in this state until diuresis commenced and then the albumen disappeared in the course of four days, as a constituent of the urine. I had before referred the anasarca to obstructed venous circulation from pressure of the uterus on the iliac veins, or perhaps upon the vena-cavae descendens itself. I accounted for its collection in the abdomen by some irritation the peritoneum might have suffered during gestation or labour, predisposing it to take on secretive action; or the fluid might have passed into the peritoneum by endosmose from the over-distended cellular tissue.

It would be very interesting to know whether there was any albumen in the urine during gestation, or during the four days immediately following labour. If this were so, we would have to refer it to the probable congestion of the kidneys by pressure of the womb upon the renal veins; while, if the albumen appeared only on the fifth day, we should refer the cause of its presence, to that condition of the system which resulted in tho untoward symptoms that then made their appearance, and the case would partake of the nature of acute disease.

Whatever explanation we may feel inclined to adopt, the interesting coincidence of a tender state of the teeth and gums with free diuresis, the rapid disappearance of the albumen from the urine before the removal of the anasarca and ascites, (forming in this respect a striking contrast with cases 2 and 3, where it was still to be found some time after all appearances of dropsy had gone) and the great improvement immediately apparent in her condition cannot fail to attract attention.

I will not say that this was salivation or that it was caused by the small quantity of calomel she took, although I think that such was the case. This sore mouth lasted a week before she could bite or eat with any comfort.

It will be seen that the last symptoms to improve, were, the pulse in loosing its rapidity and hardness, and the rheumatic pains so severe, and so rebellious to treatment.

